# Which Is the Oldest Hospital?

**Published:** 1918-05-25

**Authors:** A. H. Coughtrey

**Affiliations:** Librarian. The Library, St. Bartholomew's Hospital, London, E.C.


### THE EDITOR'S LETTER-BOX.

### Which is the Oldest Hospital?

*To the Editor of* THE HOSPITAL.

SIR,—I am afraid I must take exception to your statement that the lazar-house of St. Giles was founded before that of St. Bartholomew at Rochester. The former was founded by Queen Matilda in 1101, whereas the latter dates from 1078. I am interested in your claim for the continuity of the St. Giles institution. It should considerably widen the scope of the inquiry, and bring to light similar contentions. There can be little doubt that lazar-houses were in existence in these islands at a very early period, and it is quite possible that existing institutions may have preserved a link with some of these.—Yours faithfully,

A. H. COUGHTREY, Librarian.

The Library,
St. Bartholomew's Hospital, London, E.C.
May 18, 1918.

